# Sellar reconstruction without intrasellar packing after endoscopic surgery of pituitary macroadenomas is better than its reputation

**DOI:** 10.3205/000234

**Published:** 2016-06-23

**Authors:** Mostafa Ismail, Abd Alla Fares, Balegh Abdelhak, Jean D’Haens, Olaf Michel

**Affiliations:** 1 Department of Otorhinolaryngology, University Hospital Brussels, Vrije Universiteit Brussels, Brussels, Belgium; 2 Department of Otorhinolaryngology, Minia University Hospital, Minia University, Minya, Egypt; 3 Department of Radiology, University Hospital Brussels, Vrije Universiteit Brussels, Brussels, Belgium; 4 Department of Neurosurgery, University Hospital Brussels, Vrije Universiteit Brussels, Brussels, Belgium

**Keywords:** transsphenoidal surgery, pituitary macroadenomas, sellar reconstruction, intrasellar packing, CSF leakage

## Abstract

**Objectives:** Sellar reconstruction with intrasellar packing following endoscopic resection of pituitary macroadenomas remains a subject of clinical and radiological discussion particularly, when an intraoperative cerebrospinal fluid (CSF) leakage is absent. This study was conducted to contribute our experience with sellar reconstruction after a standard endoscopic surgery of pituitary macroadenomas without intraoperative CSF leakage to the ongoing discussion between techniques with and without intrasellar packing.

**Methods:** A consecutive series of 47 pituitary macroadenomas undergoing excision via a standard endoscopic endonasal transsphenoidal surgery (EETS) without evident intraoperative CSF leakage were retrospectively evaluated over a 10-months mean follow-up period. According to the sellar reconstruction technique, three groups could be identified: Group A – with no intrasellar packing, Group B – with haemostatic materials packing, and Group C – with abdominal fat packing. Postoperative clinical and radiological assessments of the three groups were documented and analyzed for differences in outcome.

**Results:** Postoperative clinical assessment did not differ significantly between the three groups. In group A, postoperative CSF leakage, sphenoid sinusitis and empty sella syndrome were not observed. However, a significant difference in radiological assessment could be identified; the interpretation of sellar contents in postoperative MRI of group A succeeded earlier and more reliably than in other groups with intrasellar packing.

**Conclusions:** There is no difference in the incidence of postoperative CSF leakage and empty sella syndrome among the various reconstructive techniques with and without intrasellar packing, irrespective of size and extension of the pituitary adenoma. Sellar reconstruction without intrasellar packing following a standard EETS is not inferior to other techniques with packing and even shows more radiological advantages, which made it our preferred technique, at least if no intraoperative CSF leakage is evident.

## Zusammenfassung

**Ziele:** Die Rekonstruktion der Sella mit intrasellärer Tamponade nach endoskopischer Resektion von Hypophysenmakroadenomen bleibt in der klinischen und radiologischen Diskussion umstritten, besonders wenn keine Liquorfistel vorhanden ist. Diese Studie wurde durchgeführt, um unsere Erfahrungen mit der Sellarekonstruktion nach einer endoskopischen Standardresektion von Hypophysenmakroadenomen ohne intraoperative Liquorfistel zur laufenden Diskussion über Techniken mit und ohne intrasellärer Tamponade beizutragen.

**Methoden:** Eine konsekutive Serie von 47 Hypophysenmakroadenomen, die in einem chirurgischen Standardverfahren endoskopisch über einen endonasalen transsphenoidalen Zugang ohne ersichtliche intraoperative Liquorfistel entfernt wurden, wurde nachträglich während einer durchschnittlich zehnmonatigen Nachbeobachtungszeit ausgewertet. Bezüglich der sellären Rekonstuktionstechnik konnten drei Gruppen identifiziert werden: Gruppe A – ohne intraselläre Tamponade, Gruppe B – mit Tamponade aus hämostatischen Materialien und Gruppe C – mit Bauchfetttamponade. Die postoperative klinische und radiologische Bewertung der drei Gruppen wurde dokumentiert und auf Unterschiede in den Ergebnissen hin analysiert.

**Ergebnisse:** Die postoperative klinische Beurteilung ergab keine signifikanten Unterschiede zwischen den drei Gruppen. In der Gruppe A wurden postoperativ weder Liquorfistel noch Keilbeinhöhlenentzündung oder Empty-Sella-Syndrom beobachtet. Allerdings konnte ein signifikanter Unterschied in der radiologischen Bewertung identifiziert werden: die Interpretation von sellären Inhalten bei der postoperativen MRT der Gruppe A gelang früher und zuverlässiger als in anderen Gruppen mit intrasellärer Tamponade.

**Schlussfolgerung:** Es gibt keinen Unterschied in der Inzidenz von postoperativen Liquorfisteln und Empty-Sella-Syndromen bei den verschiedenen rekonstruktiven Techniken mit und ohne intrasellärer Tamponade, unabhängig von Größe und Ausdehnung des Hypophysenadenom. Die Sellarekonstruktion ohne intraselläre Tamponade, die während eines chirurgischen Standardverfahrens endoskopisch über einen endonasalen transsphenoidalen Zugang durchgeführt wird, ist anderen Techniken mit Tamponade nicht unterlegen und bietet zudem Vorteile bei der radiologischen Diagnostik, was sie zu der von uns bevorzugten Technik macht, solange offensichtlich keine intraoperative Liquorfistel vorliegt.

## Introduction

Reconstruction of a skull base defect is essential for restoring anatomy [1], [2], [3] and for creating a watertight barrier between the sinonasal and intracranial cavities [4] in particular, following the standard and extended endoscopic endonasal transsphenoidal surgery (EETS). The routine reconstruction of sella turcica has been followed for many decades using either intrasellar autologous or synthetic packing materials [5] to prevent CSF leakage and empty sella syndrome [6].

Following a standard endoscopic surgical resection of pituitary macroadenomas without encountered intraoperative CSF leakage, a minimal packing of the extradural intrasellar space using haemostatic materials [3], [4], [5], [7], [8] or abdominal fat graft in cases having an overt thin diaphragma sellae [3], [7] is mostly appreciated for more surgical assurance. 

Intraoperative CSF leakage is considered the main predictor for the occurrence of postoperative CSF leakage [3], [5], [9], [10]. Therefore, a technique without intrasellar packing following a standard EETS of pituitary macroadenomas could provide a lesser morbidity for the patient and an unequivocal interpretation of postoperative MRI readings, if not increasing the frequency of postoperative CSF leakage or empty sella syndrome [6], [11]. 

The purpose of this study was to validate the appropriateness and effectiveness of omitting intrasellar packing following a standard endoscopic resection of pituitary macroadenomas without an evident intraoperative CSF leakage no matter size and extension of the tumour.

## Materials and methods

### Patient population

Between 2013 and 2015, 93 patients harbouring pituitary adenomas underwent consecutive surgical resection via an endoscopic endonasal transsphenoidal surgery (EETS) by one neurosurgeon (J. D.) at University Hospital (Vrije Universiteit Brussels). The Institutional Review Board of The University Hospital of Vrije Universiteit Brussels approved this study. 

Patients diagnosed with pituitary macroadenomas, undergoing standard EETS and without evident intraoperative CSF leakage (n=47) were included in current series. Patients diagnosed with pituitary microadenomas, macroadenomas undergoing extended EETS (extended transtuberculum, transplanum approach) or patients with evident intraoperative CSF leakage were excluded from the analysis.

The current series consisted of 24 women and 23 men. Patients’ ages ranged from 16 to 84 years, with a mean of 50 years. In all surgeries, an ear, nose and throat (ENT) surgeon (M. I.) was present for analysing workflow and for documentation. All patients were routinely subjected to the standard preoperative clinical, endocrinal and radiological assessments.

According to maximum tumor diameter; current series included 10 small macroadenomas (21.3%) with a maximum diameter ranged from >10 mm to <20 mm and 37 large macroadenomas (78.7%) with a maximum diameter ≥20 mm. In 24/37 large macroadenomas a suprasellar extension (SSE) was detected. They were determined according to a modified Hardy’s classification [12] into 2 grades; moderate SSE up to 10 mm (Grade A, n=15) and large SSE up to 20 mm (Grade B, n=9). 

### Surgical procedure

In all patients undergoing standard EETS, rigid endoscopes 300 mm in length and 4 mm in diameter (Olympus, Hamburg, Germany) with angled lenses of 0°, 30°, and 70° were used. A pneumatically powered holder with smooth control of the joints (Aesculap, Tuttlingen, Germany) made it possible to reposition the endoscope instantly during surgery. The complete technique was described by D’Haens et al. [13]. 

After a cross-shaped opening of the dura, adenomas were removed using a piecemeal technique. Thereupon, resection of the suprasellar components were achieved following its spontaneous descent into the pituitary fossa in most cases, facilitated by enhancement of the intracranial venous pressure by Valsalva maneuver in few cases. Extended transsphenoidal approaches were not required in any case of the current series. At the end of the procedure, the integrity of the diaphragm sellae and arachnoid membrane was inspected for CSF leakage using angled endoscopes.

**Figure 1:**
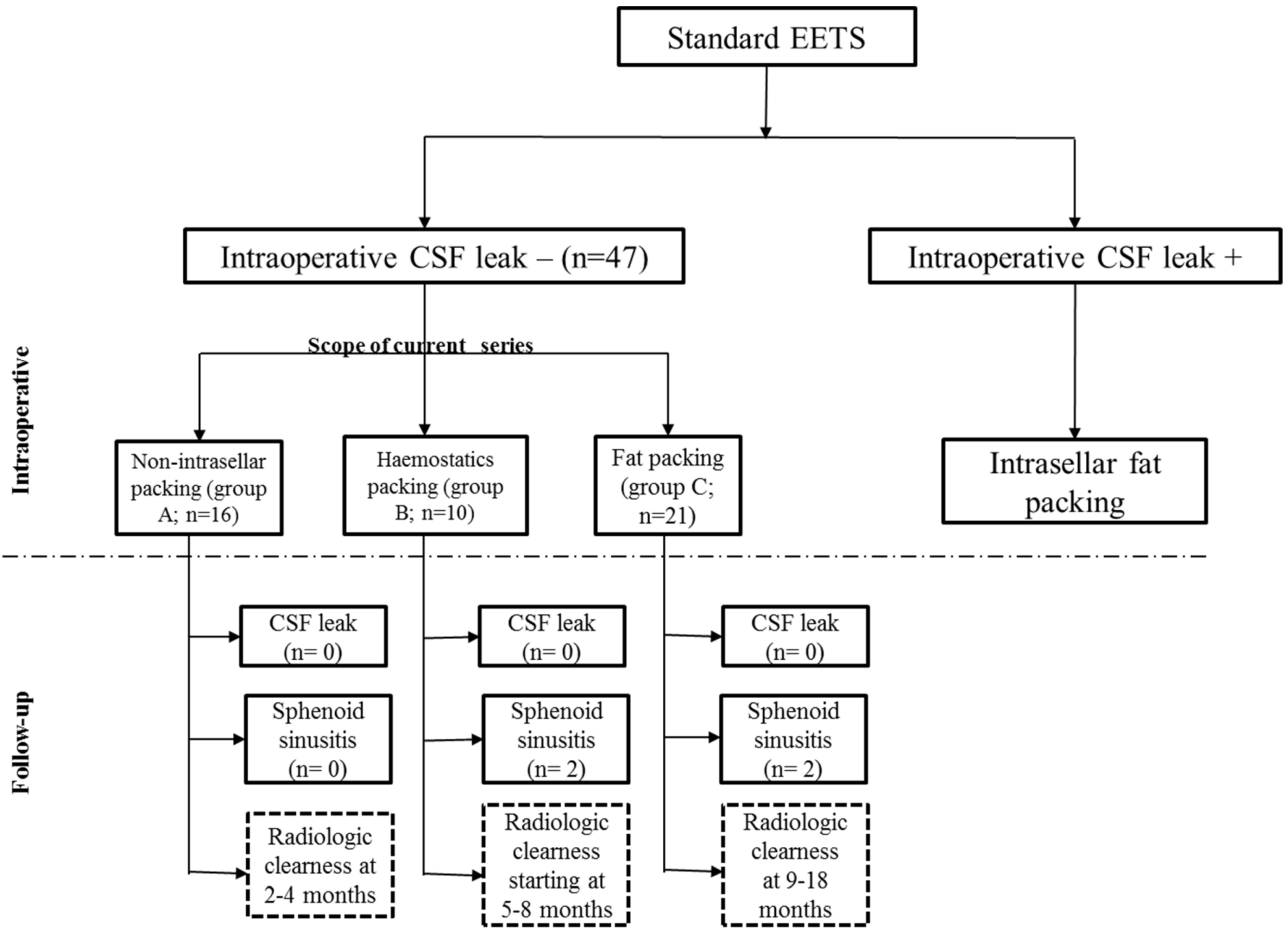
Algorithm for sellar reconstruction after standard endoscopic endonasal transsphenoidal surgery (EETS)

All patients underwent sellar reconstruction at the completion of the procedure based on the algorithm expressed in Figure 1 (Fig. 1). The sellar floor was always remodeled using bone fragments from the sphenoid rostrum and fibrin glue at the end of surgery. When an intraoperative CSF leakage was evident, intrasellar abdominal fat packing was performed; those cases were excluded from current series. 

The presented series started, in the absence of intraoperative CSF leakage, with extradural intrasellar fat packing for sellar reconstruction as in the resection of large macroadenomas, in particular, cases with thinned or prolapsed diaphragm sellae. With growing experience, supported by positive postoperative results, the reconstruction shifted gradually to an intrasellar packing using haemostatic materials only (Gelfoam or Surgicel) and even less. At the end of the observation period the classic intrasellar packing techniques as used before were completely abandoned in favor of keeping the sellar fossa empty without any packing. 

Therefore, according to the type of sellar reconstruction, three groups could be distinguished in the presented series: group A with no intrasellar packing materials, group B with haemostatic materials packing and group C with abdominal fat packing (Table 1 (Tab. 1)).

### Postoperative assessment

All patients (n=47) underwent postoperative clinical and radiological assessment by MRI (Table 2 (Tab. 2)). In patients with unremarkable clinical and radiological findings, a short follow-up was adopted.

Clinical assessment included detailed history and endoscopic examination for detection of postoperative CSF leakage and/or sphenoid sinusitis as well as documentation of any postoperative visual deterioration.

With successful surgical cure or disease control, MRI scans were routinely obtained 2–4 months (n=18) or 5–8 months (n=12) following surgery. However, in cases with lack of clinical and/or endocrinal response or with possible surgical complications (n=14), earlier postoperative studies after 1 month may be necessary. Thereafter, follow-up MRI scans were performed between 9–18 months (n=15). Six MRI scans were obtained at 24 months after surgery.

In order to compare the radiological appearance of the pituitary fossa after different sellar reconstruction techniques, with and without packing materials, all postoperative MRI scans were evaluated. This was done in regard of the radiological interpretation of sellar contents and the occurrence of radiological empty sella with assessment of optic chiasm in the early, late and delayed postoperative follow-up. 

### Statistical analysis

All the data was analyzed using Statistical Package of Social Sciences (SPSS) v20.0 for Microsoft Windows 7. Outcome variables were analyzed by a simple categorical frequency comparison with the Chi-square (χ^2^). The ANOVA test was used to compare the three groups. P<0.05 was considered statistically significant.

## Results

### Clinical assessment

After a mean 10-months follow-up period (range: 2 to 24 months); no significant differences were found between the three groups in postoperative clinical assessment regarding the assessed parameters; CSF leakage, sphenoid sinusitis and visual deterioration.

Sellar reconstruction without intrasellar packing was followed in a total 16 macroadenomas in group A; 9 of them classified harbouring SSE (56%), with no evident early or late postoperative CSF leakage. This was compared to both groups with intrasellar packing. No postoperative CSF leakage was experienced following surgical resection of all macroadenomas in group B and C; under them 5 and 10 cases with SSE were recorded respectively. No patient experienced early or late postoperative visual deterioration in the three groups after a mean of 10 months follow-up.

In both groups B and C, 4 patients developed postoperative sphenoid sinusitis, in spite of a routinely wide sphenoidotomy technique done in all presented cases. No sphenoid sinusitis was recorded in group A. Postoperative sphenoid sinusitis was diagnosed clinically by persistent headache and postnasal discharge in all patients. MRI of the 4 patients showed opacified sphenoid sinuses along with dropped fat content in one of them. All patients restored by medication except one patient from group B who required sinus surgery after 19 months. 

### Radiological assessment

**Figure 2:**
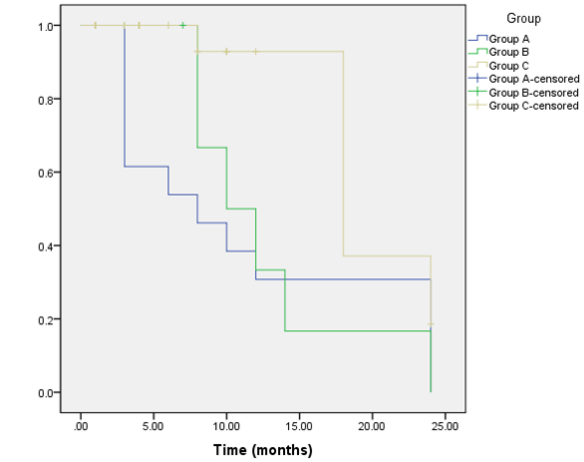
MRI findings regarding clear radiological interpretation of sellar contents in the three groups

Radiological assessment of the pituitary fossa regarding the adequate interpretation of sellar contents (defined as clear identification of normal pituitary and residual tumors tissues after complete absorption of postoperative changes and packing materials), gave significant different results in the three groups in the late (2–4 months) and delayed (9–18 months) MRI examinations (p=0.001 and 0.036; Figure 2 (Fig. 2)). 

**Figure 3:**
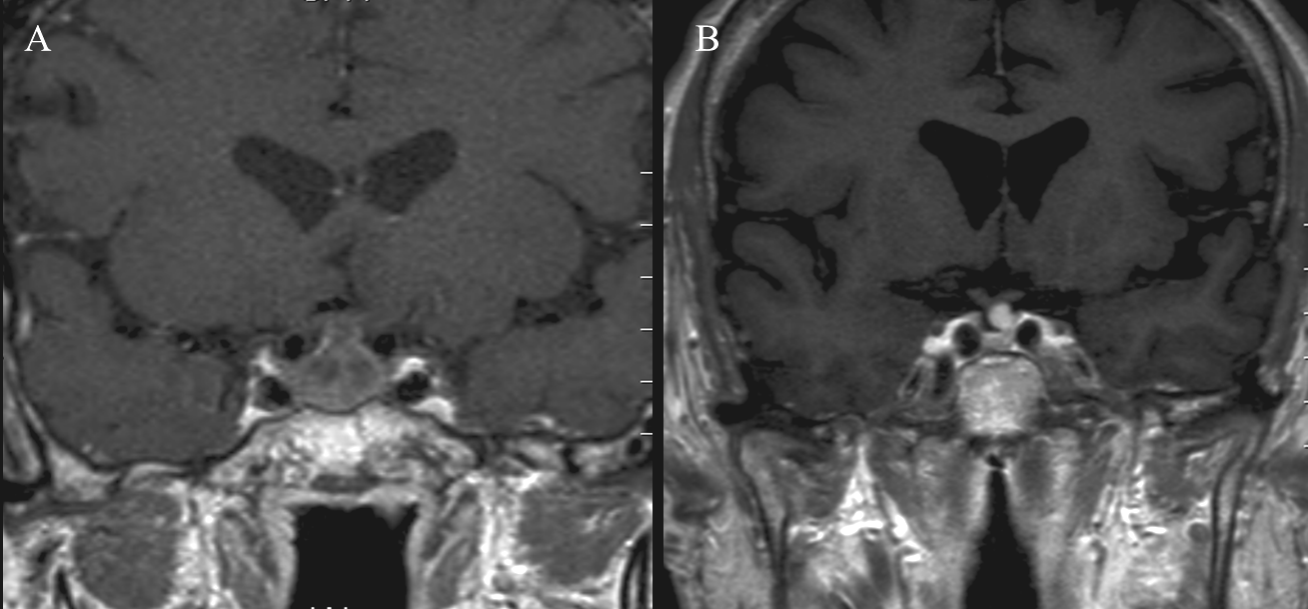
Coronal T_1_-weighted contrast enhanced MRI images of one patient from group A: A) Preoperative image showing large macroadenoma with moderate suprasellar extension (SSE). B) Postoperative image at 3 months showing complete absorption of postoperative changes with proper interpretation of the remaining pituitary tissue.

In group A, in all early examinations at 1 month (n=3); opacification of the pituitary fossa was noticed, for which blood and inflammatory exudate were radiologically considered responsible. All the late and delayed examinations (n=13) showed complete disappearance of the postoperative changes giving the possibility for adequate interpretation of the sellar contents (Figure 3 (Fig. 3)).

**Figure 4:**
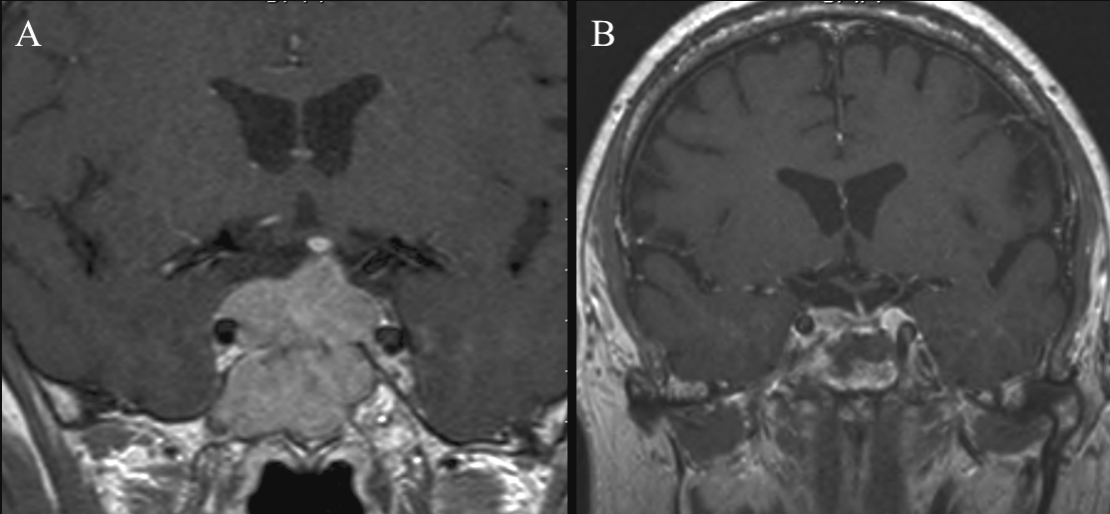
Coronal T_1_-weighted contrast enhanced MRI images of one patient from group B: A) Preoperative image showing large macroadenoma with moderate suprasellar extension (SSE), intra-sphenoid sinus invasion and suspected right cavernous sinus invasion. B) Postoperative image after 3 months showing iso-intense area on right side with a circumscribed rim of enhancement compatible with residual adenoma which could not be differentiated from haemostatic packing materials.

In group B, the gradually degraded haemostatic materials, starting after 2 months, along with formation of a contrast-enhancing granulation tissue, in the period from 3–8 months which detected in (n=4) images, were hold responsible for difficult interpretation of sellar contents in (n=5 images; 71%) of the late examinations (Figure 4 (Fig. 4)). All delayed examinations (n=4) showed complete disappearance of the opacifying elements. 

**Figure 5:**
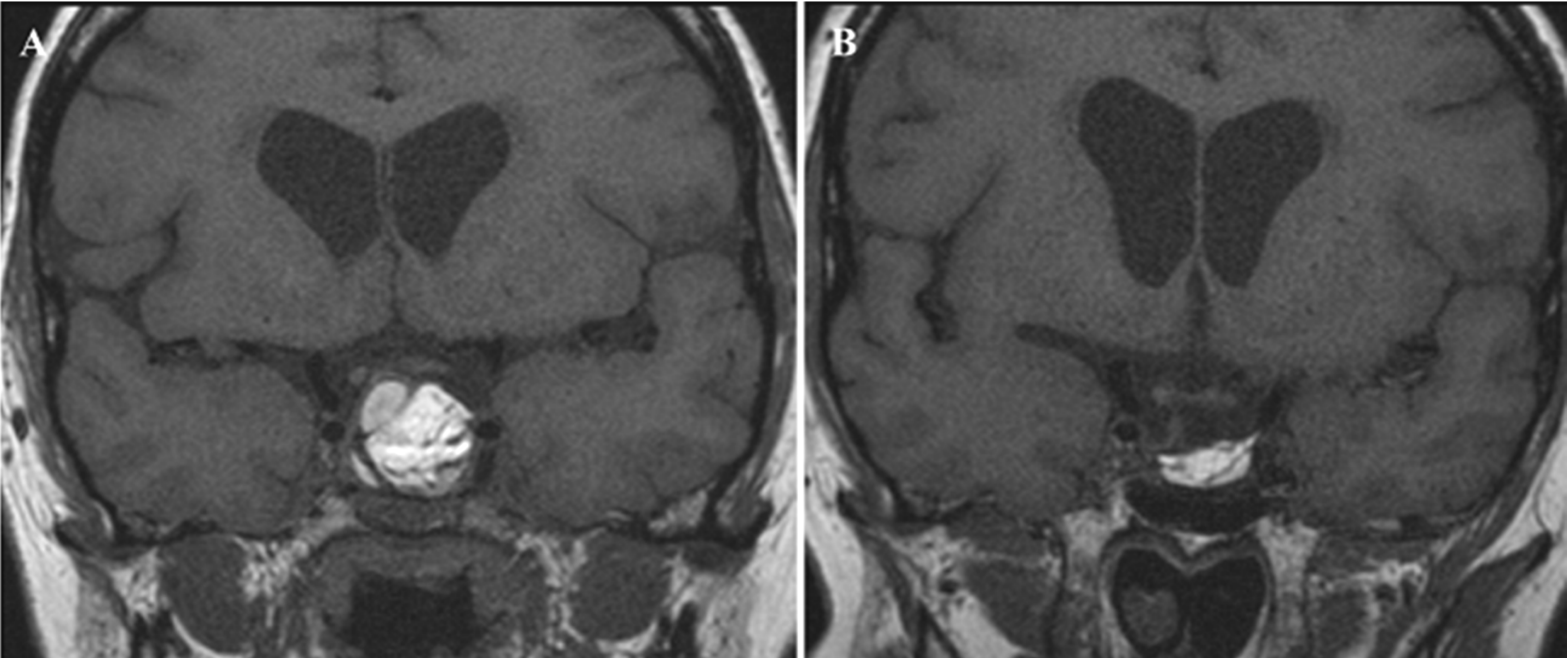
Postoperative T_1_-weighted coronal MRI images of one patient from group C: A) At 3 months showing opacification of the pituitary fossa with hyper-intense fat signal protruding inside sphenoid sinus in addition to an area with mixed iso- and hypo-intensity on right and superior part of the pituitary fossa, suspected residual adenoma. B) The same patient after 10 months showing reduced size of intrasellar fat interlaced with iso-intense streaks of fibrous tissue with complete disappearance of the area with suspected residual adenoma.

In group C, fat tissue could be identified as long as 8 to 18 months after surgery. In one case, it was evident as late as 24 months postoperatively. Fatty tissues have a hyper-intense signal and could be easily detected in all images. Delayed absorption of postoperative changes up to 4 months after surgery, in addition to formation of an iso-intense fibrous tissue in the period from 5–18 months, were responsible for inadequate interpretation of sellar contents in all examinations in the first 4 months (n=19) and 69% (n=11) of the examinations performed later (Figure 5 (Fig. 5)).

Downward displacement of optic chiasm was recorded in two patients; one from group A and one from group C following resection of macroadenomas with suprasellar extensions. Radiological empty sella was recorded in the same patient from group A.

## Discussion

Endoscopic endonasal transsphenoidal surgery (EETS) has been predestined and evolved since its incipient description as a minimal invasive technique for treatment of sellar lesions. However, sellar reconstruction is still under discussion due to the variability of techniques and materials used [7]. The routine reconstruction of the sella turcica with intrasellar packing has been performed using either autologous materials such as fascia, muscle, and fat or synthetic materials [5] including Gelfoam and collagen fleece [9] with the purpose to prevent CSF leakage and empty sella syndrome [6].

Although the presence of an intraoperative CSF leakage has been proved as the main predictor factor for the occurrence of postoperative CSF leakage [3], [5], [9], [10], sellar reconstruction with intrasellar packing has been the unspoken standard even when an intraoperative CSF leakage has not been evident following standard EETS of pituitary macroadenomas. In cases with preoperative and/or intraoperative risk factors for postoperative CSF leakage and empty sella syndrome such as large macroadenomas [14], [15], suprasellar extension (SSE) [16], or cases with thinning or prolapsing diaphragma sellae [3], [7], still intrasellar packing using one or two layers of collagen sponge [3], [4], [5], [7], [8] or abdominal fat graft [3], [7] is recommended for more surgical assurance. 

The presented study proves again that intrasellar packing is not indispensable if CSF leakage is not evident intra-operatively; irrespective of size and extension of the resected pituitary macroadenomas. In the hereby analysed series of 47 pituitary macroadenomas including large macroadenomas with SSE (n=24, 51%), no postoperative CSF leakage was observed after standard EETSs, whatever the type of sellar reconstruction used; with or without intrasellar packing. 

Sphenoid sinusitis after EETS for sellar lesions is a rare postoperative complication because of the routinely performed wide sphenoidotomy obviating outlet obstruction of the sphenoid sinus orifice. Postoperative sphenoid sinusitis was not recorded in the group without intrasellar packing compared to both groups with intrasellar packing in which sphenoid sinusitis was equally recorded in 4 patients. This may be ascribed to the absence of haemostatic or autologous packing materials in group A which may trigger tissue reaction and obstruction of the sphenoidotomy outlet [17].

Although the diaphragma sella with elongated optic nerves and optic chiasm are prone to herniate into sella after resection of large cystic or soft tumours, acute visual deterioration rarely develops in this situation [6]. There is no correlation between the presence of downward displacement of optic chiasm and the visual disturbances [18], [19].

In our series we did not record any case with postoperative visual deterioration, despite downward displacement of optic chiasm in postoperative MRI of two patients from group A and C. This was comparable to the experience of Chen and Lee [6] who recorded a radiological empty sella in 2 patients without intrasellar packing and one patient with intrasellar fat packing. However, no visual deteriorations were recorded. Also Steiner’s et al. [20] findings in optic chiasm herniation, which was observed in one-third of the patients, showed no visual disturbances. 

Intrasellar packing materials may represent also a radiological challenge in the assessment of postoperative MRI [21] due to delayed postoperative clearance of packing materials, intrasellar blood and inflammatory exudate [22]. As known from the literature, haemostatic materials can be detected on MRI in the first month [21] or even late up to 3–6 months [20], [23]. It was detected up to 4 months in our series. After gadolinium contrast administration, haemostatic packing materials tend to show peripheral rim of enhancement caused by granulation tissue [20], [21], [23]. This usually starts at 3 months postoperatively [23] and may be visible up to 8 months, according to our series. All these changes may affect the adequate identification of sellar contents and residual tumours. 

Implanted fat has, due to its persistence, a longer lasting intrasellar effect [24] up to 12 months [6]. In our series it was observed up to 8 to 18 months postoperatively. Although identification of fat in MRI examination is simple due to its characteristic high signal intensity [21] and a possible use of fat-suppression sequences, it may interfere with the radiological identification of the sellar contents [16], [25]. 

Together with all the reported observations, our results give more evidence that the suspicion of complications such as CSF leakage and symptomatic empty sella syndrome are not necessarily consequence when omitting intrasellar packing after standard EETS of pituitary macroadenomas. Furthermore pursuing omitting intrasellar packing materials has contributed to more precise and earlier radiological interpretation of sellar contents after complete absorption of the postoperative changes. This growing positive experience has been an incentive for us to keep the intrasellar space free without packing materials if no intraoperative CSF leakage is encountered.

### Limitations of the study

Our study implies some restrictions being a retrospective study with a single institutional study design and a shortened follow-up period of some patients because of logistical and patient-related issues.

These data, even if arising from a modest patient sample, underlines the appropriateness and effectiveness of omitting intrasellar packing following a standard endoscopic resection of pituitary macroadenomas without an evident intraoperative CSF leakage. Randomized controlled perspective studies may follow.

## Conclusion

Our presented observations underline the concept that in absence of an intraoperative CSF leakage following a standard EETS of pituitary macroadenoma, the omission of intrasellar packing materials is possible and can be followed successfully without increase in postoperative morbidities such as CSF leakage, sphenoid sinusitis and symptomatic empty sella syndrome regardless of size and extension of the resected macroadenoma. As an additional radiological advantage, it provides earlier adequate interpretation of sellar contents and reliable assessment of residual tumour.

## Notes

### Competing interests

The authors declare that they have no competing interests.

## Figures and Tables

**Table 1:**
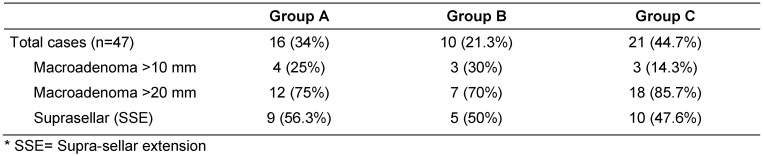
Summary of the preoperative data for the three groups

**Table 2:**
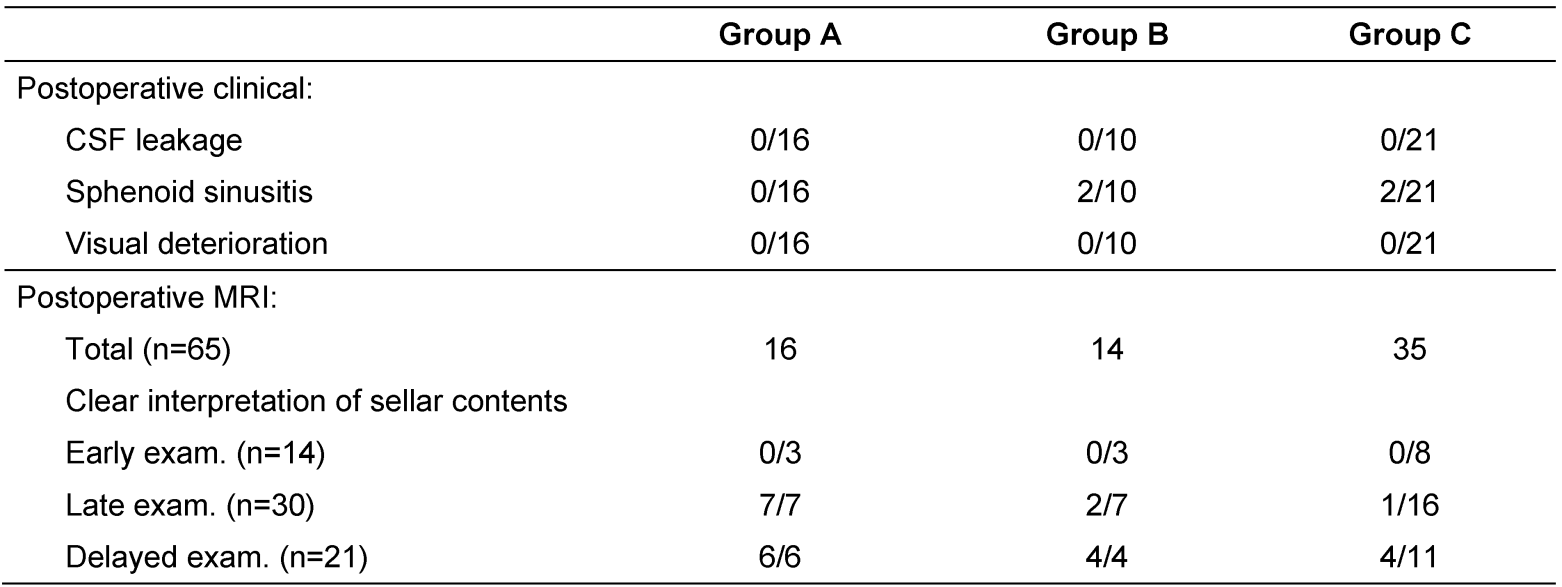
Summary of the postoperative clinical and radiological results for the three groups
